# Evaluating cell lines as tumour models by comparison of genomic profiles

**DOI:** 10.1038/ncomms3126

**Published:** 2013-07-09

**Authors:** Silvia Domcke, Rileen Sinha, Douglas A. Levine, Chris Sander, Nikolaus Schultz

**Affiliations:** 1 Computational Biology Center, Memorial Sloan-Kettering Cancer Center, 1275 York Avenue, Box 460, New York, New York 10065, USA; 2 Department of Chemistry, Technische Universität München, Lichtenbergstraße 4, 85747 Garching bei München, Germany; 3 Department of Surgery, Memorial Sloan-Kettering Cancer Center, 1275 York Avenue, New York, New York 10065, USA; 4 These authors contributed equally to this work

## Abstract

Cancer cell lines are frequently used as *in vitro* tumour models. Recent molecular profiles of hundreds of cell lines from The Cancer Cell Line Encyclopedia and thousands of tumour samples from the Cancer Genome Atlas now allow a systematic genomic comparison of cell lines and tumours. Here we analyse a panel of 47 ovarian cancer cell lines and identify those that have the highest genetic similarity to ovarian tumours. Our comparison of copy-number changes, mutations and mRNA expression profiles reveals pronounced differences in molecular profiles between commonly used ovarian cancer cell lines and high-grade serous ovarian cancer tumour samples. We identify several rarely used cell lines that more closely resemble cognate tumour profiles than commonly used cell lines, and we propose these lines as the most suitable models of ovarian cancer. Our results indicate that the gap between cell lines and tumours can be bridged by genomically informed choices of cell line models for all tumour types.

## Abstract (web summary)

Cell lines are widely used in cancer research to study tumour biology. Here Domcke *et al.* compare genomic data from ovarian cancer cell lines with those from clinical ovarian tumour samples and identify cell lines that most closely resemble the genomic features of high-grade serous ovarian cancer.

## Graphical abstract

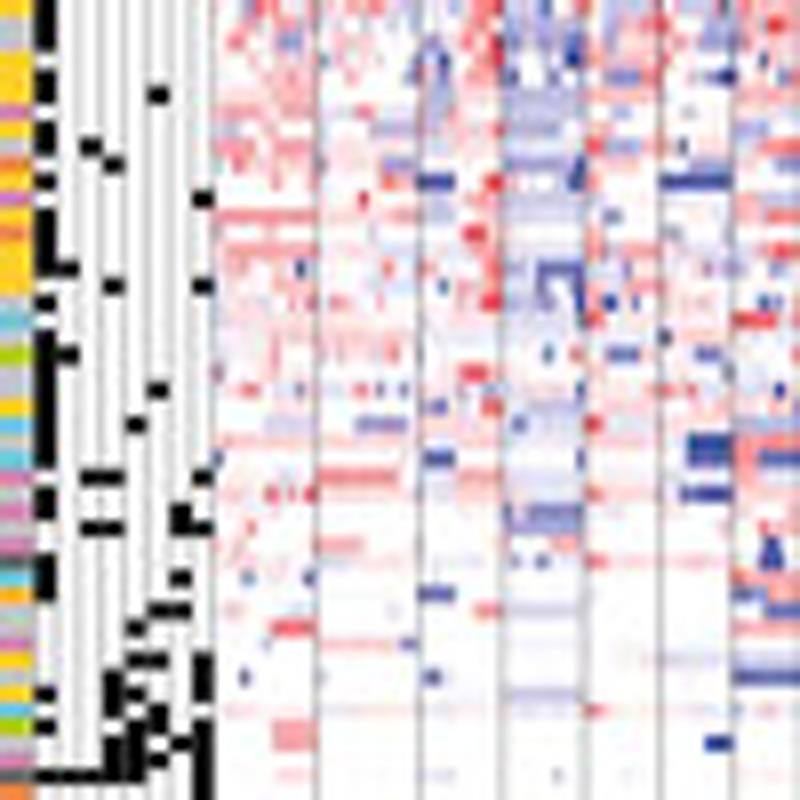

Cell lines derived from tumours are the most frequently utilized models in cancer research and their use has advanced the understanding of cancer biology tremendously over the past decades. Genomic differences between cancer cell lines and tissue samples have been pointed out in several studies^1^,^2^,^3^,^4^. However, owing to the lack of large-scale genomic data, finding the cell lines that most closely resemble the genomic alterations of a given tumour (sub)type has been difficult. Now for the first time, a large set of molecular profiles are available for both tumour samples and cell lines: In The Cancer Genome Atlas (TCGA), the genomes and expression profiles of at least 500 tissue samples per tumour type are being comprehensively characterized^5^. The Broad-Novartis Cancer Cell Line Encyclopedia (CCLE) contains genomic profiles of around 1,000 cell lines that are used as models for various tumour types^6^. These efforts enable a systematic comparison of tumours and cell lines at the level of DNA copy-number, mutation and mRNA expression data across a diversity of tumour types. In this pilot study, we focus on high-grade serous ovarian cancer (HGSOC) and seek to identify the ovarian cancer cell lines most suitable as *in vitro* models based on comparison of the available genomic profiles.

Every year, >100,000 women around the globe die of ovarian cancer^7^. In the United States, ovarian cancer is the most lethal gynaecological malignancy and fifth leading cause of cancer death for women^8^.

Epithelial ovarian cancer is traditionally divided into four major histological subtypes: serous, endometrioid, clear cell and mucinous carcinoma. Serous ovarian carcinoma is responsible for ~70% of epithelial ovarian cancers^9^. The most aggressive subtype, HGSOC, accounts for 90% of these serous carcinomas^10^ and two-thirds of all ovarian cancer deaths^11^, making it by far the most extensively studied ovarian carcinoma.

Until recently, all histological subtypes were believed to arise from the ovarian surface epithelium and were often not differentiated in preclinical research or clinical trials. However, the discovery that the majority of invasive tumours may stem from different non-ovarian tissues accompanied by molecular analysis of the respective subtypes has led to the recognition that ovarian cancer is extremely heterogeneous and in fact comprises several distinct diseases^12^,^13^.

The most commonly used cell line models for ovarian cancer—and implicitly for the most prevalent subtype HGSOC—are SK-OV-3, A2780, OVCAR-3, CAOV3 and IGROV1 (quantified via Pubmed citations, see Results). However, their histopathological origin is partly unclear, and the need for well-characterized cell lines as models for the respective subtypes of ovarian cancer has been repeatedly voiced^12^,^13^.

Our comparison of data from TCGA and the CCLE reveals striking differences between some of the most commonly used cell line models and the majority of HGSOC samples. On the basis of our findings, we recommend an alternative set of ovarian cancer cell lines more suitable for *in vitro* studies of HGSOC. Although conclusions based on *in vitro* cell line experiments are not necessarily valid in a clinical setting, choosing cell lines most representative of certain subtypes should increase the value of cell line studies in preclinical research.

## Results

### Genomic characterization of HGSOC

**Figure 1:**
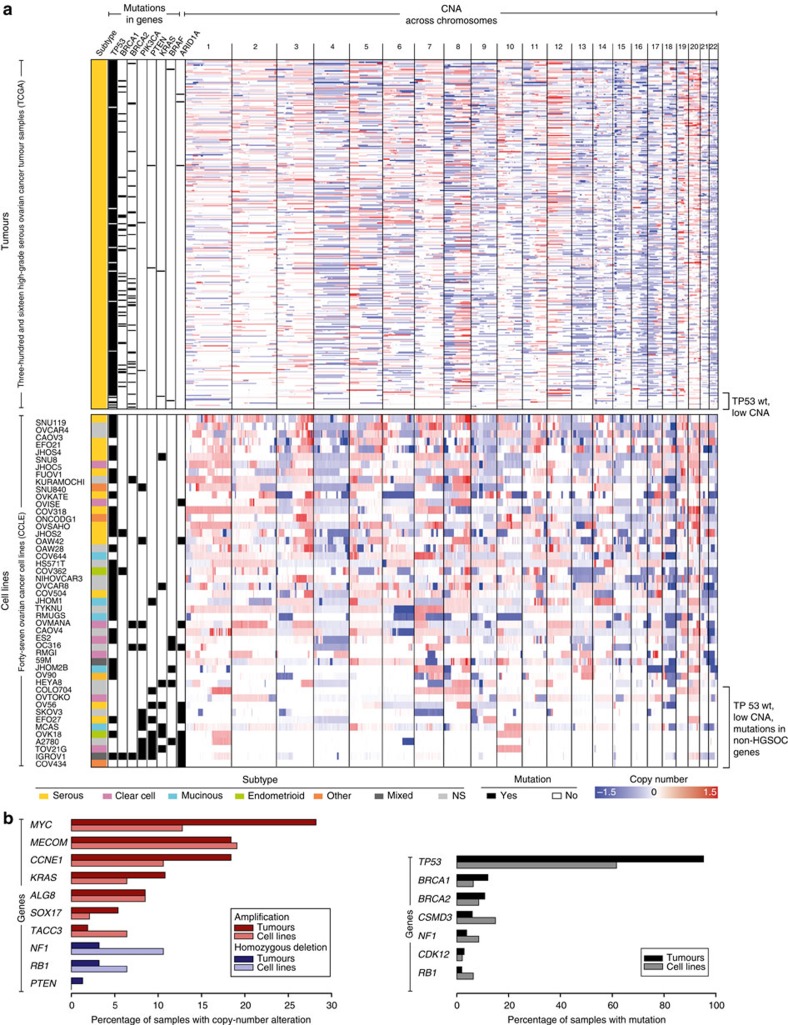
Genomic comparison of TCGA HGSOC samples with CCLE ovarian cancer cell lines suggests overall genomic similarity. (**a**) CNA profiles (right, chromosomes 1–22) and mutations (left, in eight selected genes) of HGSOC patient samples from TCGA, top and ovarian cancer cell lines from the CCLE, bottom. The samples are sorted according to decreasing fraction of the genome altered in DNA copy number. Somatic mutations in genes known to be commonly altered in one of the four epithelial ovarian cancer subtypes are indicated on the left, with germline mutations included for *BRCA1* and *BRCA2* in the tumour samples in addition to the somatic mutations. Note the samples with a low degree of CNA coinciding with wild-type *TP53* copies near the bottom of each panel (square bracket). (**b**) The most frequent genomic alterations identified in HGSOC tumour samples and their occurrence in the ovarian cancer cell line panel: CNAs (left) and mutations (right).

The TCGA study revealed three major genomic features of HGSOC. First, copy-number alterations (CNAs) are remarkably common in HGSOC, with the median fraction of the genome altered as large as 46% (Fig. 1a, Supplementary Fig. S1A). Second, *TP53* mutations are near universal (95% of samples), and the few tumours with wild-type *TP53* predominantly have flat copy-number profiles (Fig. 1a). Third, the overall frequency of somatic mutation in protein-coding regions is low, with only *TP53*, *BRCA1* and *BRCA2* mutated in >10% of samples (Fig. 1b)^5^.

These features set the HGSOC subtype apart from the low-grade serous, endometrioid, clear cell and mucinous ovarian carcinomas, which have near-normal gene copy-numbers and wild-type *TP53* (refs 14, 15, 16). Comprehensive information on protein mutations in these other subtypes of ovarian cancer is not yet available, but the known mutations differ strongly from the mutation spectrum of HGSOC. For instance, two-thirds of low-grade serous carcinomas carry mutations in *KRAS*, *BRAF* or *ERBB2* (refs 17, 18, 19). Low-grade endometrioid carcinoma is characterized by *ARID1A* mutations in one-third of the tumours^20^, as well as *CTNNB1* mutations^21^, *PTEN* mutations^22^ and *PIK3CA* mutations^23^. *ARID1A* mutations are similarly found in nearly half of clear cell carcinomas^20^, and *PIK3CA* mutations are common^23^. The majority of mucinous carcinomas are mutated in *KRAS* (ref. 18).

Interestingly, some of the HGSOC tumour samples profiled in TCGA with wild-type *TP53* have mutations in one of the genes typically altered in non-HGSOC subtypes, as well as uncharacteristically flat copy-number profiles (Fig. 1a), casting doubt on their origin. Histopathological reassessment of these tumour samples should reveal whether they truly belong to the HGSOC or rather a different ovarian cancer subtype. In an independent collection, HGSOC samples with wild-type *TP53* in fact showed diverse histology after pathological review or evidence of *TP53* dysfunction^24^, implying that loss of *TP53* function is truly universal in HGSOC.

### Comparison of cell lines and tumour samples

At first glance, the CCLE ovarian cancer cell line panel appears to have overall genomic similarity to the HGSOC tissue samples. On the DNA copy-number level, the median fraction of the genome altered in the 47 ovarian cancer cell lines in the CCLE data set is quite similar to that of the TCGA tumours, although the distribution is wider for the cell line panel (Fig. 1a, Supplementary Fig. S1A). This is not surprising, given that the CCLE data set encompasses diverse subtypes of ovarian cancer, which are known to differ drastically in their copy-number status^16^. The most frequent CNAs in TCGA are all represented to some extent among the CCLE ovarian cancer cell lines (Fig. 1b). The most recurrently mutated genes in HGSOC are also mutated in a considerable fraction of the cell lines (Fig. 1b): *TP53* is mutated in 62% of cell lines, and *BRCA1* and *BRCA2* in 6% and 9%, respectively.

However, closer inspection reveals substantial differences between some of the cell lines and the tumours. In general, more mutations were identified in the cell lines in the 1651 genes profiled in both studies (median frequency of 4.3 per Mb in cell lines versus 1.6 per Mb in tumours; Supplementary Fig. S1B). Several factors plausibly contribute to the larger number of mutations reported for the cell line panel. First, cell lines are purer than tumour samples, which tend to be contaminated with stromal cells. Second, apart from *BRCA1* and *BRCA2,* the TCGA study only considers somatic mutations, whereas the mutations identified in the CCLE also include private germline variants. Third, mutations acquired during *in vitro* culturing are a further possible contributing factor.

Apart from general differences between cell lines and tumours, some of the cell lines in the panel further differ from the HGSOC tumour samples because they probably originate from other, non-HGSOC subtypes. For example, *PIK3CA* is mutated in 19% of the ovarian cancer cell lines but in <1% of TCGA HGSOC samples (Fig. 1a). Although mutations in *PIK3CA*, *KRAS*, *PTEN*, *BRAF* and *ARID1A* are uncommon in HGSOC, they are characteristic of other ovarian cancer subtypes. Strikingly, a higher mutation frequency in one of these ‘non-HGSOC’ oncogenes or tumour suppressors tends to coincide with a flat copy-number profile and wild-type *TP53* (Fig. 1a), making these cell lines possible models of low-grade serous, endometrioid, clear cell or mucinous ovarian carcinoma^17^,^18^,^19^,^20^,^21^,^22^,^23^,^24^,^25^,^26^.

### Five ovarian cancer cell lines are hypermutated

**Figure 2:**
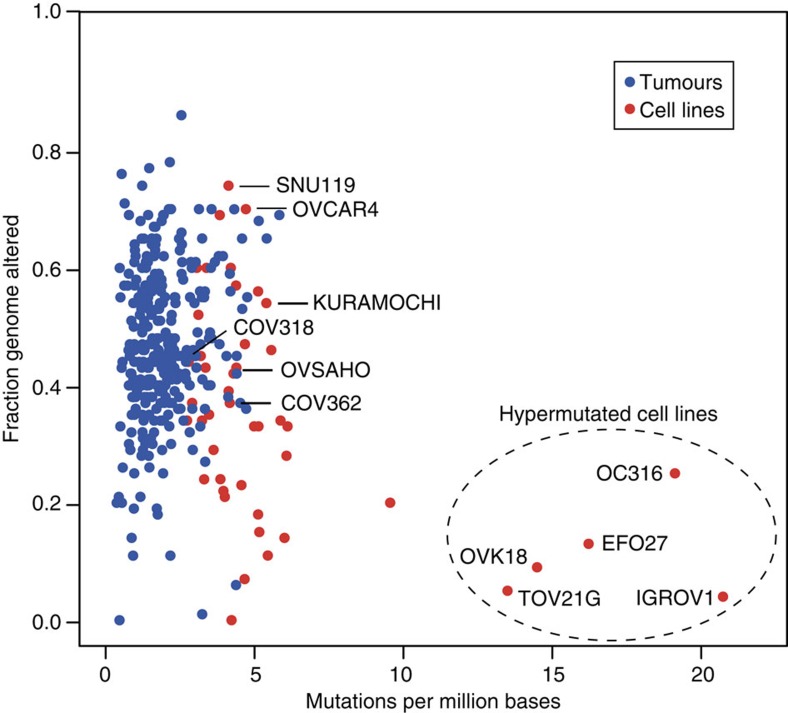
Hypermutated cell lines are outliers. The comparison of mutation frequency (horizontal) and degree of CNA (vertical) for HGSOC tumour samples (blue) and ovarian cancer cell lines (red) reveals a subset of cell lines (dashed ellipse) with a hypermutator genotype (high mutation frequency, few DNA copy-number changes). The hypermutated cell lines (mutation frequency in parentheses) are: IGROV1 (20.7/Mb), OC316 (19.0/Mb), EFO27 (16.1/Mb), OVK18 (14.4/Mb) and TOV21G (13.4/Mb). Cell lines that on the contrary resemble the tumour samples in key characteristics (as shown below in Fig. 3) are also labelled.

Although the ovarian cancer cell lines typically have a slightly higher number of mutations than HGSOC tumour samples, they have a similar degree of CNAs (Fig. 2). However, five cell lines are outliers with opposite characteristics: IGROV1, OC316, EFO27, OVK18 and TOV21G not only have few CNAs but also surprisingly many mutations. This ‘hypermutator’ genotype sets them clearly apart from the rest of the ovarian cancer cell lines and from the HGSOC tissue samples.

### Key genomic features of suitable cell line models of HGSOC

**Figure 3:**
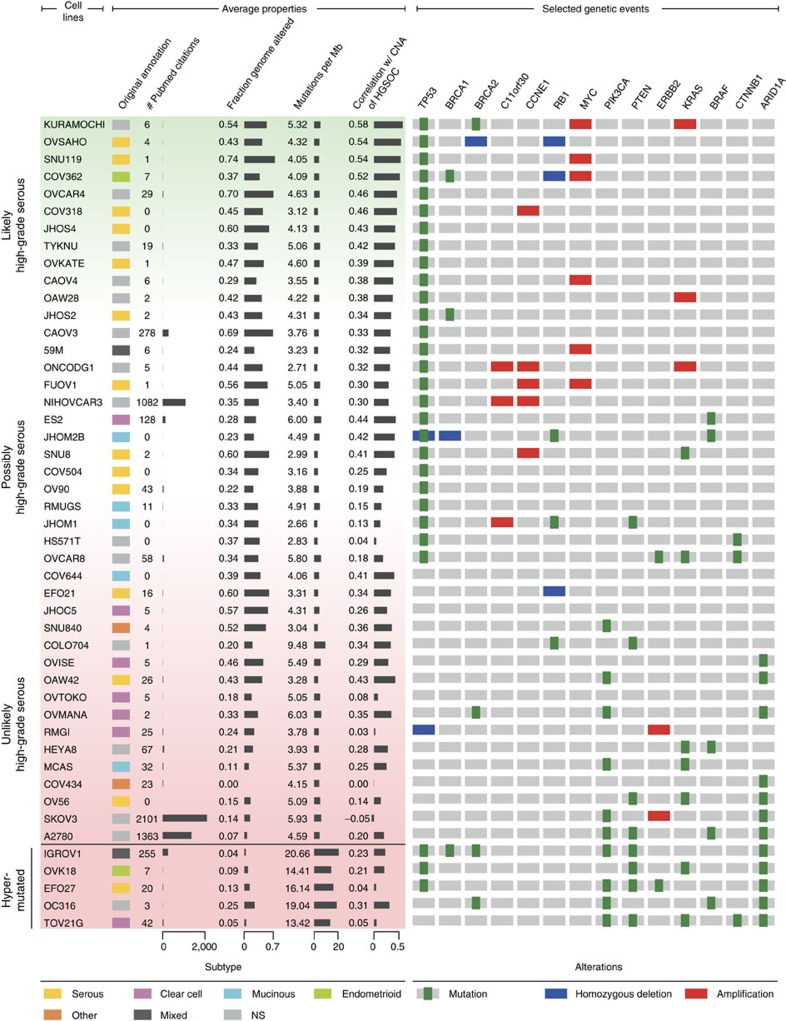
Ranking ovarian cancer cell lines by suitability as HGSOC models. Both average properties (left) and selected genetic events specific to ovarian cancer (right) can be used to distinguish better and poorer models of HGSOC. Average properties include the histological subtype as determined in the original publication (references in Supplementary Data S1), the citation frequency in the literature as estimator of frequency of use in laboratories, the altered fraction of the genome, the number of mutations per million bases and the correlation with the mean CNA profile of HGSOC tumour samples. The selected genetic events include alterations recurrently found either in HGSOC (mutation of *TP53*, *BRCA1* or *BRCA2*; amplification of *C11orf30* (*EMSY*), *CCNE1*, *KRAS* or *MYC*; mutation or deletion of *RB1*) or one of the three other major subtypes of ovarian cancer (mutation in *PIK3CA*, *PTEN*, *KRAS*, *BRAF*, *CTNNB1* or *ARID1A*; mutation or amplification in *ERBB2*). The colour gradient underlying the cell line names to the left indicates better (green) versus poorer (red) models of HGSOC according to selected characteristics (*TP53* status, correlation with mean CNA profile of TCGA samples, low mutation rate and absence of mutations in the seven ‘non-HGSOC’ genes, see Supplementary Data S1). The hypermutated cell lines described in Fig. 2 are located at the bottom of the table. Note that although HGSOC cell lines are probably at the top and unsuitable cell lines are at the bottom of the table (vertical labels), the order does not signify an exact ranking of cell line models.

Although the altered fraction of the genome and total mutation count can reveal clear outliers among the cell lines, these criteria are summary properties and not sufficient for identifying appropriate tumour models. For a more detailed gene-by-gene comparison of copy-number data, we calculated the correlation of the CNA profile of each cell line to the tumours (see Methods, Supplementary Data S1). For identifying cell lines resembling the majority of HGSOC tumour samples, the correlation with the mean CNA of tumours is of most interest. This mean CNA profile takes into account amplifications or deletions consistently present in many samples, whereas conflicting or noisy copy-number values are averaged out (Fig. 3, left).

In addition to these average properties, alterations in cancer genes known to have a specific functional role in diverse cancer subtypes can help distinguish more or less suitable cell line models (Fig. 3, right). To best discriminate between HGSOC and other ovarian cancer subtypes, we chose, on one hand, alterations characteristic of HGSOC, such as mutations in *TP53* and *BRCA1/2* as well as amplifications in *C11orf30* (*EMSY*), *CCNE1*, *MYC*, *PIK3CA* and *KRAS* (ref. 5) and, on the other hand, mutations in a subset of genes recurrently altered by mutation only in other ovarian cancer subtypes (*PIK3CA*, *PTEN*, *ERBB2*, *KRAS*, *BRAF*, *CTNNB1* and *ARID1A*).

### Ranking of cell lines by suitability as HGSOC models

To evaluate the suitability of any particular cell line as a model for HGSOC tumours, we defined a set of plausible criteria. Although these criteria are not exhaustive, nor tailored to particular research questions, they can be a reasonable guide to avoid clearly unsuitable cell lines and choose those that at least resemble tumour samples in terms of overall and specifically functional criteria. We thus divided the 47 ovarian cancer cell lines into good, moderate and poor models of HGSOC using an empirical numerical score (see Methods). The suitability score is higher (1) the better the correlation between the copy-number profile of the cell line and the mean copy-number profile of HGSOC tumour samples; (2) the lower the frequency of non-synonymous mutations in protein-coding genes; (3) in the presence of a *TP53* mutation; and (4) in the absence of mutations in the seven ‘non-HGSOC’ genes (see above) commonly altered in other ovarian cancer subtypes (Fig. 3, Supplementary Data S1). Applying the score leads to a reasonable ordering of the cell lines from the most suitable (top, green, Fig. 3) to the least suitable (bottom, red, Fig. 3). This order is useful for selecting or deselecting cell lines, but is not meaningful as a finely graduated ranking.

### Good and bad cell line models

The grouping by suitability score in Fig. 3 provides a guide to cell line selection. The cell lines near the top feature the major genomic characteristics of HGSOC, and thus seem best suited as *in vitro* models for HGSOC. These cell lines have a *TP53* mutation but no mutation in the seven non-HGSOC genes. Their copy-number profiles correlate well with the mean CNA of all tumours. They also have a high correlation with the copy-number profile of a single tumour sample (Supplementary Data S1), and their alteration pattern in the ovarian cancer-specific gene set matches the TCGA samples. Strikingly, the twelve best candidates in this analysis account for only 1% of current Pubmed citations out of the 47 analysed cell lines, although HGSOC is by far the most prevalent and extensively studied ovarian cancer subtype (Fig. 3, Supplementary Fig. S2).

Of the dozen cell lines with the highest suitability score, many were indeed classified as serous cell lines by the pathologists in the original publications (Fig. 3). For a sizeable group, the histological subtype was not or could not be specified in the original publication. Among these is the cell line with the highest suitability score, KURAMOCHI: its copy-number profile highly correlates with the mean CNA of HGSOC tumours and the copy-number profile of a single tumour, it has a low mutation frequency and HGSOC-specific alterations in key oncogenes and tumour suppressors (Fig. 3, Supplementary Data S1). In the original publication, however, KURAMOCHI is rather ambiguously classified as undifferentiated carcinoma^25^,^26^. As this cell line has all the major characteristics of HGSOC, our analysis implies that it was in fact derived from this tumour subtype. Interestingly, a cell line classified as endometrioid in the original publication, COV362, is among the top-ranking HGSOC-like cell lines^27^. Although this is surprising at the first sight, high-grade endometrioid carcinomas are in fact difficult to distinguish from HGSOC at the morphological and molecular level^28^. As these tumours also have high CNA and mutations in *TP53* (ref. 16), it has been recently suggested that they actually belong to the HGSOC subtype^12^,^29^. Taken together, these observations highlight that the subtypes assigned to cell lines at their derivation based on histopathology are not necessarily identical to their molecular subtypes.

Although several cell lines resemble HGSOC tumour samples, there are also several cell lines that have little resemblance to HGSOC and a low suitability score (Fig. 3, bottom), among them the hypermutated cell lines mentioned above. Most of these poorly matched cell lines were not classified as high-grade serous by the pathologists in the original publication. The lack of HGSOC features in these cell lines stemming from ovarian carcinomas of the endometrioid, clear cell or mucinous subtype can be explained by the substantial molecular differences between the diverse subtypes of ovarian cancer^17^,^18^,^19^,^20^,^21^,^22^,^23^,^24^,^25^,^26^. However, among the low-ranking cell lines, there are also some that were classified as serous in the original publication. Low- and high-grade serous carcinomas were not differentiated in most of the original publications. Today these subtypes are recognized as different diseases with diverse genomic profiles^20^,^21^,^22^. Some of the low-ranking cell lines whose parent tumours were described as serous but have only modest CNA and an uncharacteristic mutation profile, therefore plausibly stem from low-grade serous tumours. Again, this suggests that insights from molecular profiles can help to refine the traditional histopathological annotation of subtypes.

All cell lines were derived at least 13 years ago and have been in passage for a considerable time. A substantial number was derived not directly from primary tumours in the ovary but rather from ascitic fluid or peritoneal deposits (Supplementary Data S1). Interestingly, no correlation was observed between the time of derivation of a cell line (as substitute measure for passage number) or the specimen site and the estimated suitability as tumour model.

For some preclinical studies, cell lines with *BRCA* mutations are of particular interest, given the implication of this gene in the prevention and treatment of HGSOC^5^,^12^,^30^. The fraction of *BRCA* mutation carriers lies at roughly 10% for both the ovarian cancer cell line panel and the HGSOC tumour samples (Fig. 1b). However, out of the six cell lines with a *BRCA* mutation, two are among the hypermutated cell lines (IGROV1, OC316) and one has wild-type *TP53* and uncharacteristic mutations (OVMANA). However, the top-ranking HGSOC-like cell line, KURAMOCHI, as well as two further cell lines (COV362, JHOS2) also carry *BRCA* mutations, and therefore constitute possible models for *in vitro* investigation of ‘BRCAness’ in HGSOC (Fig. 3).

### Popular cell line models do not closely resemble HGSOC tumours

SK-OV-3, A2780, OVCAR-3, CAOV3 and IGROV1 are the most popular cell line models as quantified by Pubmed citations, accounting for 90% of publications mentioning at least one of the 47 CCLE ovarian cancer cell lines (Supplementary Fig. S2). Although the exact histological origin is not specified in the original reference for most of them, they are commonly used as models for HGSOC. OVCAR-3 and CAOV3 possess *TP53* mutations and substantial copy-number changes, key characteristics of HGSOC. However, they are not among the top-ranking HGSOC-like cell lines owing to a lower correlation value with the mean CNA as well as lower correlation values with the CNA of individual tumours (Fig. 3, Supplementary Data S1). Strikingly, the two most frequently used cell lines, SK-OV-3 and A2780, which together account for 60% of publications on this cell line panel, are poorly suited as models for HGSOC. Both have a very flat copy-number profile, and they do not have *TP53* mutations but instead mutations frequently found in other histological subtypes, such as *ARID1A*, *BRAF*, *PIK3CA* and *PTEN* mutations. This lack of HGSOC characteristics stands in stark contrast to the frequent use of these cell lines as models for this subtype.

### IGROV1 is most probably not of the HGSOC subtype

IGROV1 is often quoted as being of the HGSOC subtype^31^,^32^,^33^,^34^,^35^,^36^,^37^,^38^,^39^,^40^,^41^,^42^,^43^. However, its flat copy-number profile and high mutation frequency place it among the hyper-mutators described above (Figs 1a, 2 and 3). The large number of mutations is most probably due to frameshift mutations in the DNA repair genes *MLH1*, *MSH3* and *MSH6*. Similar loss of *MLH1* or *MSH2* expression has been observed in endometrioid cancers^44^. With frameshift mutations in *ARID1A*, an activating missense mutation in *PIK3CA* (R38C) (ref. 45) and an inactivating missense mutation in *PTEN* (Y155C) (ref. 46), IGROV1 not only has the overall genomic profile but also several specific signature mutations of endometrioid carcinoma. Especially, the co-occurrence of *PIK3CA* and *PTEN* mutations is rare in general but has been described in both endometrial and endometrioid carcinomas^15^,^47^.

**Figure 4:**
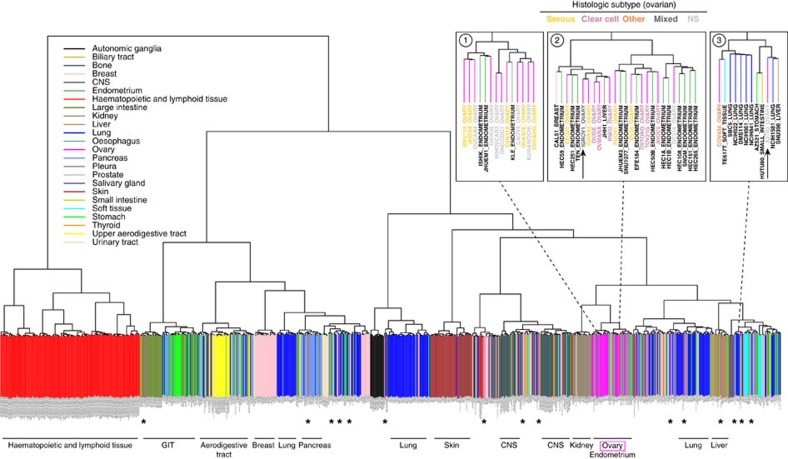
Expression-based clustering of all 963 CCLE cell lines from diverse tumour types. The 5,000 most variable genes were used for unsupervised clustering of cell lines by mRNA expression data. Cell lines are colour-coded (vertical bars) according to the reported tissue of origin (a PDF version that can be enlarged at high resolution is in Supplementary Information, Supplementary Fig. S4); horizontal labels at bottom indicate the dominating tissue types within the respective branches of the dendrogram. Most ovarian cancer cell lines (magenta) cluster together, interspersed with endometrial cell lines. However, some ovarian cancer cell lines cluster with other tissue types (*). Top right panels: neighbourhoods (1) of the top cell lines in our analysis, (2) of cell line IGROV1, and (3) of cell line A2780. For the ovarian cancer cell lines in these enlarged areas, the histological subtype as assigned in the original publication is indicated by coloured letters.

Expression profiles of tumours and cell lines were compared to further corroborate our observations made on the copy-number and mutation level. We computed the correlation of the expression profiles of all cell line and tumour pairs, and ranked the cell lines by the average of their correlations with the tumours. The correlation between this ranking and the ordering produced by the suitability score assigned based on copy-number and mutation data is highly significant (*P*-value 1.27e−05, Kendall’s tau rank-correlation test; Supplementary Data S2). Clustering both the ovarian cancer cell lines and the HGSOC tumours based on expression data is not as informative of the relative suitability of the 47 cell lines as tumour models, as a clear division between cell lines and tumours is observed, both by unsupervised clustering as well as by principal component analysis (Supplementary Fig. S3)^48^. Expression-based clustering of all CCLE cell lines from all tumour types, however, groups most cell lines according to their tissue of origin, thus providing valuable information (Fig. 4)^49^. Interestingly, IGROV1 clusters with endometrial and clear cell ovarian cancer cell lines. In light of the recent discovery linking both endometrioid and clear cell ovarian cancers to endometriosis, this observation is no longer surprising^20^. Taken together, these findings imply that IGROV1 is of endometrioid or clear cell rather than high-grade serous origin. In fact, the original publication describes the parent tumour as mainly endometrioid carcinoma with serous, clear cell and undifferentiated areas^50^.

### Expression clustering suggests diverse tissues of origin

IGROV1 is not the only cell line that has acquired an inaccurate subtype label in the literature. The field has come to realize that several ovarian tumours in fact do not originate in this organ but rather constitute metastases stemming from distant primary tumours^12^. Interestingly, several CCLE ovarian cancer cell lines cluster with non-ovarian cancer types by mRNA expression data (Fig. 4, Supplementary Data S1). Among these is A2780, the second most commonly used ovarian cancer cell line. By expression, it clusters far from the majority of ovarian cancer cell lines with the lung, liver, stomach and small intestine cancer cell lines, and its copy-number and mutation profiles show no resemblance to the TCGA samples (Figs 1a, 3 and 4).

Some cell lines are not classified as HGSOC, although they have all hallmarks of this cancer subtype. An especially striking example of this is KURAMOCHI, which is one of the top HGSOC-like cell lines in the above analysis and clusters with serous ovarian cancer cell lines in the expression data set. Indeed, the top-ranking HGSOC-like cell lines in terms of CNA and mutation patterns all cluster together in the expression data analysis (Fig. 4). These cell lines, assigned a high suitability score based on their genomic features, therefore also share somewhat similar mRNA expression profiles, further corroborating that they stem from the same tissue type, that is, HGSOC tumours.

In short, several cell lines considerably resemble HGSOC with respect to copy-number, mutation and expression data. On the other hand, three cell lines commonly used as models for this subtype, namely SK-OV-3, A2780 and IGROV1, have little profile similarity to the tumours.

## Discussion

Several publications have recently pointed out the need for good cell line models of the distinct subtypes of ovarian cancer and especially the most prevalent HGSOC^12^,^13^. Which cell line is the optimal tumour model depends on numerous factors such as the problem at hand, the specific genomic alterations of interest as well as more practical issues like growth characteristics, and thus has no single answer. However, for certain studies, such as drug sensitivity assessment, maximal molecular similarity to tissue samples is desirable. Our analysis can serve as a general guideline for choosing appropriate and avoiding poorly suited cell line models of HGSOC.

Alarmingly, this study reveals that the most frequently used cell lines seem for the most part badly suited for investigating HGSOC, whereas the cell lines that more closely resemble the tumours are rarely used in laboratories. Indeed, the dozen top-ranking HGSOC-like cell lines account for only 1% of Pubmed citations out of the 47 analysed cell lines, although HGSOC is by far the most prevalent and extensively studied ovarian cancer subtype. Although limited commercial availability could have contributed to the infrequent use of some of the top-ranking HGSOC cell lines, it cannot fully explain it. The top HGSOC-like cell lines are all obtainable from one of the major commercial distributors^6^. Another plausible reason for the striking discrepancy between suitability and frequency of use of cell lines is the ambiguity of subtype annotations of cell lines in the literature.

For several cell lines, the subtype assumed in publications is not mirrored by the molecular profiles. The most striking example, IGROV1, has a hypermutator genotype and is possibly of endometrioid or clear cell origin. Although the original publication describes mainly the endometrioid nature of the parent tumour, over the years the subtype annotation of IGROV1 in publications has evolved to HGSOC. Further examples of miscommunication in the literature are the most frequently used ovarian cancer cell lines A2780 or SK-OV-3, which were not assigned any histological subtype by the originators, but today are widely assumed to be good models of HGSOC. On the other hand, there are cell lines like KURAMOCHI or OVCAR-4, which are not as frequently used and could not be assigned a histological subtype by the originators, but whose genomic features place them among the HGSOC cell lines. Taken together, these issues raise the question of potential composite use of classical histopathology and genomic profiling for subtype identification of parent tumours but also of the derived cell lines. Especially, when the histopathological diagnosis is ambiguous, it may be advisable to complement visual microscopic classification by quantitative evaluation of genomic attributes, which should soon be available to pathology at reasonable cost.

Cell line models for the distinct cancer subtypes that are clearly annotated and whose identity has been confirmed by a combination of targeted sequencing and copy-number profiling or single nucleotide polymorphism-fingerprinting can be particularly valuable in the clinic, especially in the age of personalized medicine. On one hand, preclinical results, for example, measurement of drug response profiles, obtained in well-characterized cell line models with known alterations may be a very useful guide to patient selection in clinical trials at a level of subdivision that would lead to higher response rates. On the other hand, in light of the advances in molecular profiling, one can envision the reverse scenario: for a given patient, determine the molecular profile of the tumour, select the most similar cell line model by means of a more refined suitability score, use this cell line to perform preclinical drug screens and as a result make a more informed choice of therapy for the patient. A more practical and straightforward form of cell line selection as *in vitro* models of patient tumours could already be implemented today. The realization that the distinct subtypes of ovarian cancer may be diverse diseases has prompted calls for distinguishing between these subtypes in clinical trials. Taking another step back, it is reasonable to use cell lines of the same subtype as the intended patient cohort in preclinical studies. There are examples of failed clinical trials conducted in HGSOC patients after preclinical studies in cell lines of endometrioid origin, among them IGROV1 (ref. 31). It is not guaranteed that using cell line models of the same subtype would have influenced the preclinical results. However, using cell lines with genomic background similar to patient samples at least increases the likelihood that conclusions reached in an *in vitro* setting will be transferable to the clinic. Although several of the cell lines analysed here are genomically similar to HGSOC, deriving new cell lines from untreated primary ovarian tumours will probably help to further bridge the gap between cell line models and clinical tumours. The cell lines profiled by CCLE have been in passage for several years, if not decades, and some patients were treated with severe chemotherapy before the biopsy, both factors that are known to affect genomic profiles^51^,^52^.

In summary, in this study we distinguish ‘the good, the bad and the ugly’ among cell line models of HGSOC. We recommend a set of ‘good’ cell lines that closely resemble tumour samples (Fig. 3). In contrast, we point out several ‘bad’ cell line models of this subtype that have flat copy-number profiles, wild-type *TP53* and uncharacteristic mutations. This group includes the two most frequently used ovarian cancer cell lines SK-OV-3 and A2780. ‘Ugly’ cell line models make up a third group: these cell lines resemble HGSOC at the first sight, as they have *TP53* mutations or a substantial degree of CNA—but closer inspection reveals striking differences. For some of these ‘ugly’ cell lines, expression profiles imply they are derived from metastases from distant tissues. Others, such as IGROV1, are hypermutated and plausibly stem from a different ovarian cancer subtype.

This pilot study on HGSOC describes a methodology for selecting suitable cell lines as tumour models. Although the choice of the optimal cell line is highly context specific, our conceptual approach for identifying suitable cell line models is widely applicable. Hand in hand with the increasing availability of genomic data from studies such as the CCLE and the Sanger Cancer Cell Line project or TCGA and the International Cancer Genome Consortium, this method can be further refined and applied to a wide range of tumour types. In this way, it can help to optimize the choice of cell lines as tumour models for a broad variety of tumour types, and thus increase the value of preclinical studies.

## Methods

### Data acquisition

DNA copy-number, mutation and mRNA expression data were analysed for all 316 HGSOC tumour samples profiled by TCGA (ref. 5) and 47 ovarian cancer cell lines from the CCLE (ref. 6). For the remaining CCLE ovarian cancer cell lines, COLO684, TOV112D, OC314 and OC315, not all three data types were available from the CCLE, so they were excluded from the analysis. In our comparison, we consider all data types that are available for both studies: genome-wide DNA copy-number information, mutation data for 1,651 genes and mRNA expression profiles. Only recently, short-tandem repeat profiling revealed substantial redundancy and contamination in a different ovarian cancer cell line panel^53^. For the CCLE ovarian cancer cell line panel, however, identity was confirmed via single nucleotide polymorphism-fingerprinting^6^.

### Copy-number data processing

Segmented copy-number data obtained from the CCLE website (platform: Affymetrix SNP6) (ref. 6) and the cBio Cancer Genomics Portal ( http://www.cbioportal.org/)^54^ for the TCGA data (platform: Agilent 1M array)^5^ was used for the analysis of CNAs. Fraction genome altered (FGA) was calculated as follows:


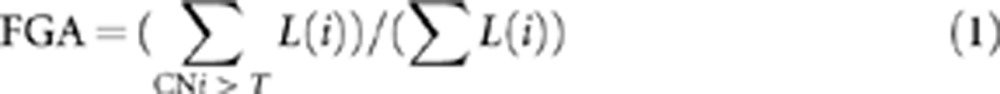


For each segment *i*, *CN*_*i*_ is given by *CN=*log2(sample intensity/reference intensity), *L(i)* is the length of segment *i* and *T* is the threshold value of the *CN*_*i*_ above which the segments are considered altered. In other words, FGA is the ratio of the sum of the lengths of all segments with signal above the threshold to the sum of all segment lengths. A threshold *T* of 0.2 was used for TCGA tumour samples and 0.3 for the CCLE cell lines. Different thresholds were chosen for the tumours and cell lines as the copy-number signal for tumours is often weakened due to contamination with non-tumour material or by tumour heterogeneity, whereas cell lines are purer. Similar reasoning was used when choosing a CN value >1.0 to define high-level amplifications in CCLE cell lines.

To enable a gene-by-gene comparison of copy-number profiles from TCGA tumour samples and CCLE cell lines, the Bioconductor package CNTools was used to map the segmented copy-number data of all CCLE and TCGA samples to genes^55^. The mean copy-number profile of the TCGA samples was obtained by computing the mean signal of each gene across all tumour samples. Correlations of copy-number profiles were calculated using Pearson’s correlation coefficients.

In detail, the similarities and differences between cell lines and tumours on the copy-number level were quantified in three different ways (see Supplementary Data S1). For each cell line, the CNA profile was compared with that of single tumours and the entire group of tumour samples. To determine similarity to single tumour samples, the correlation of the copy-number profile of each cell line with the copy-number profile of each of the 316 HGSOC tumour samples was calculated over all genes. This measure is of particular interest when seeking to identify suitable cell line models for specific subgroups of patients. On the other hand, it can be desirable to find cell lines whose copy-number profiles are most similar to those of the majority of tumour samples, disregarding any diversity within the tissue samples. To determine the similarity of the CNA of each cell line with that of the entire group of tumours, we calculated the median of all the correlation values for the 316 tumour samples. In addition, we determined the mean CNA profile of the tumour samples, that is, the copy-number change for each gene averaged over all samples. In this measure, amplifications or deletions consistently present in many samples are taken into account, whereas conflicting or noisy copy-number values are averaged out. For each cell line, the correlation of its copy-number profile with this mean CNA profile was calculated over all genes. Although these three comparisons of copy-number profiles are related, which one is most informative depends on the question at hand. Although the correlation with the mean CNA profile of tumours resembles the median of the correlations with the CNA of all single tumours for all the ovarian cancer cell lines, the correlation value with the CNA of the nearest single tumour does not necessarily follow a similar trend.

### Calculation of mutation frequencies

Mutation frequencies were calculated as the ratio of mutation counts to number of bases covered. To focus on the mutations most likely to be functional, mutations in introns, untranslated regions, flanking and intergenic regions, as well as silent and RNA mutations, were excluded. The CCLE provided the number of reads per base in the sequenced regions (in ‘wig’ format), so the number of bases covered was given by the number of positions with one or more reads. TCGA, on the other hand, provided exon-wise coverage information, namely the length of each exon and an associated coverage per exon between 0 and 1. So the effective number of bases covered for each exon was given by the product of the length and coverage of the exon. The sum of these values is the total number of bases covered for each TCGA HGSOC sample.

### Computing the cell line suitability score

The extent to which the ovarian cancer cell lines match genetic characteristics shared by the majority of TCGA high-grade serous ovarian tumours was assessed using an empirical numerical score. This suitability score *S*, in which selected features of HGSOC are positively weighted and characteristics of other ovarian cancer subtypes are negatively weighted is given by


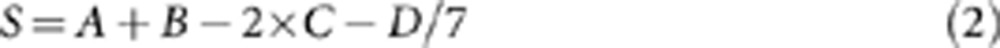


where *A* is the correlation with the mean CNA of HGSOC tumours, *B* is 1 for cell lines harbouring a *TP53* mutation and 0 otherwise, *C* is 1 for hypermutated cell lines and 0 otherwise, and *D* is the number of genes mutated among the seven ‘non-HGSOC’ genes recurrently altered only in the other ovarian cancer subtypes (*ARID1A*, *BRAF*, *CTNNB1*, *ERBB2*, *KRAS*, *PIK3CA* and *PTEN*). This score serves to distinguish better and poorer cell line models of HGSOC, but is not considered a finely graduated ranking (Supplementary Data S1).

### Expression analysis and clustering

Robust *z*-scores (median-centred expression values divided by the median absolute deviation) were used for expression-based clustering of all CCLE cell lines. The top 5,000 genes by interquartile range (difference between the 25th and 75th percentile) across all cell lines were chosen, and *1−c* (where *c* is Pearson’s correlation coefficient) was used as the distance for hierarchical clustering using Ward’s agglomeration method^56^.

For expression-based comparison of CCLE ovarian cancer cell lines and TCGA HGSOC tumour samples, *z*-scores were derived separately for the two data sets before a combined analysis was performed using the 10,383 genes available on both platforms. We used data from the Affymetrix U133A platform for TCGA, although this meant missing data for one of the 316 tumour samples, as the CCLE expression data was obtained using Affymetrix U133 Plus 2.0 Arrays. The top 5,000 genes by interquartile range across the combined data set were chosen for principal component analysis as well as hierarchical clustering using *1−c* as the distance, and complete linkage for agglomeration.

### Software tools

Data processing, analysis and visualization was done in the Perl and R programming environments, and statistical calculations were done using the R language^57^. The copy-number profiles of TCGA samples and CCLE cell lines were visualized using the Integrative Genomics Viewer (version 1.4.2)^58^ and OncoPrints were generated using the cBio Cancer Genomics Portal ( http://www.cbioportal.org)^54^. The Bioconductor package sparcl was used to draw the coloured dendrogram^55^,^59^.

### Pubmed citation analysis

The number of Pubmed abstracts mentioning one of the 47 CCLE ovarian cancer cell lines was determined using the Pubmed search builder ( http://www.pubmed.org) on 4 June 2012 using several punctuation alternatives for the cell line names. This search method can lead to false-negative results, for example, it did not yield any hits for some cell lines such as COV318, although a few publications exist that do not refer to the cell line in the abstract.

## Author contributions

S.D., R.S., D.A.L., C.S. and N.S. conceived the project. C.S. and N.S. supervised the project. S.D., R.S. and N.S. analysed and interpreted the data. S.D., R.S., C.S. and N.S. wrote the manuscript. All authors discussed the results, and reviewed and commented on the manuscript.

## Additional information

**How to cite this article:** Domcke, S. *et al.* Evaluating cell lines as tumour models by comparison of genomic profiles. *Nat. Commun.* 4:2126 doi: 10.1038/ncomms3126 (2013).

## Supplementary Material

Supplementary Figures Supplementary Figures S1-S4

Supplementary Data 1 Genomic and histologic features of 47 ovarian cancer cell lines from the Cancer Cell Line Encyclopedia. Key features determined from literature research and our analysis of molecular profiles are summarized and cell lines are sorted according to approximate suitability as models of high-grade serous ovarian cancer.

Supplementary Data 2 Ranking of 47 ovarian cancer cell lines from the Cancer Cell Line Encyclopedia based on average correlation of their expression profiles with those of high-grade serous ovarian cancer tumour samples from the Cancer Genome Atlas.
