# Supplementary material for: Evaluating cell lines as tumour models by comparison of genomic profiles
Source: Nat Commun. 2013 Jul 9;4:2126. doi: 10.1038/ncomms3126 (PMC3715866; doi:10.1038/ncomms3126)
Supplement: Supplementary Figures — S1-S4 [file ncomms3126-s1.pdf]

Figure S1

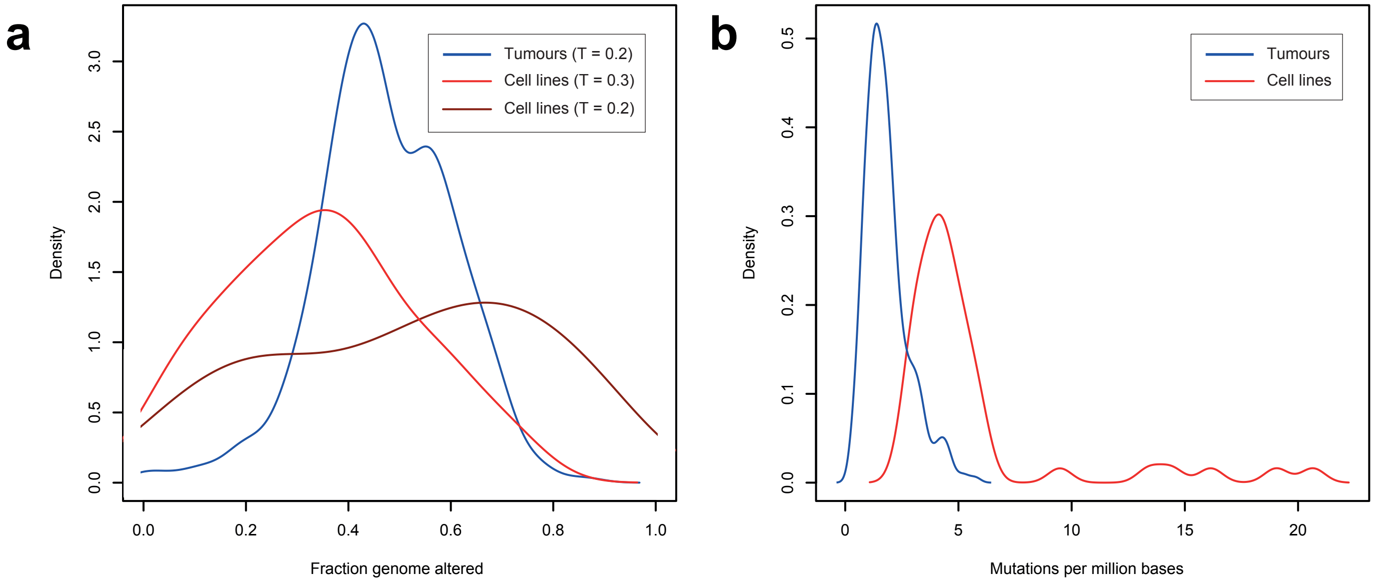

Figure S1. Comparison of overall copy-number alteration and mutation frequencies for 316 high-grade serous ovarian cancer (HGSOC) tumour samples from The Cancer Genome Atlas (TCGA) and 47 ovarian cancer cell lines from the Cancer Cell Line Encyclopedia (CCLE) indicates wider distribution and outliers in the cell line panel.
A. Fraction of the genome altered in DNA copy-number for HGSOC tumour samples (blue) and ovarian cancer cell lines (red). To account for the higher purity of cell lines and enable a more realistic comparison with tumours, a higher threshold T of the log2(sample intensity/reference intensity) above which the genome is considered altered was used for the cell lines (light red) than for the tumours (see Methods for details). The fraction of the genome altered calculated with the same threshold as used for the tumours is also shown for reference (dark red).
B. Number of mutations per million bases for HGSOC tumour samples and ovarian cancer cell lines. Mutation frequencies were calculated using both sequence and coverage data from TCGA and the CCLE.

Figure S2

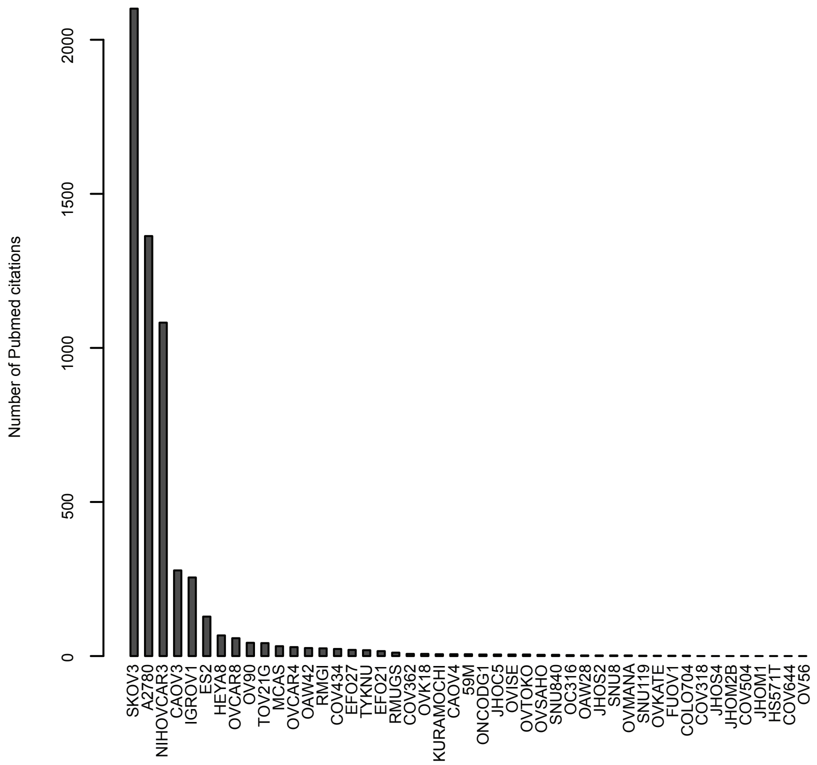

Figure S2. Number of Pubmed citations for the 47 ovarian cancer cell lines as estimator of frequency of use in laboratories.

The number of Pubmed (http://pubmed.org) abstracts mentioning one of the 47 CCLE ovarian cancer cell lines was determined with the Pubmed search builder on June 4, 2012 using several punctuation alternatives for the cell line names.

Figure S3

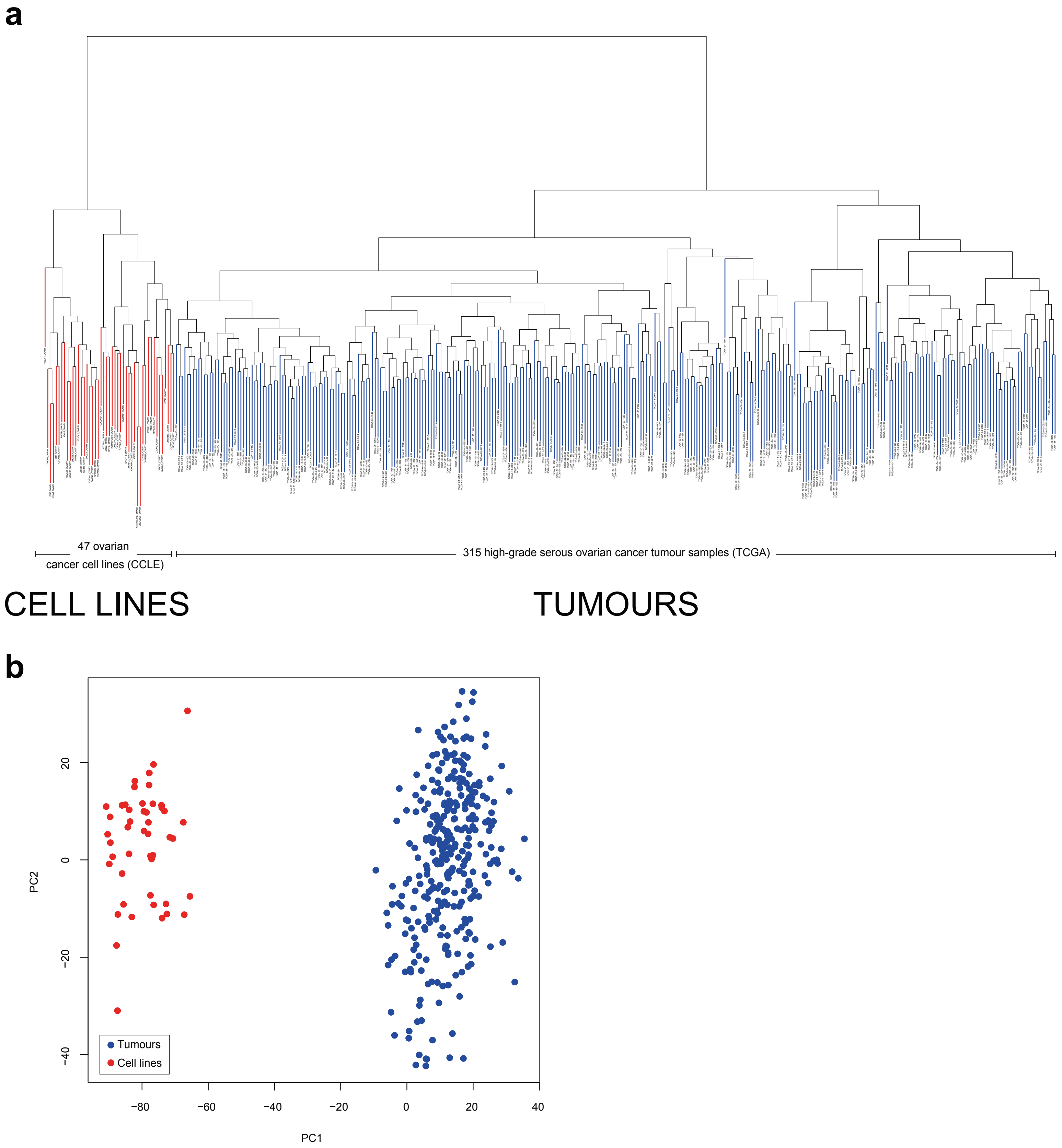

Figure S3. Ovarian cancer cell lines and high-grade serous ovarian cancer (HGSOC) tumor samples cluster apart for mRNA expression data.

A. Expression-based clustering of 47 ovarian cancer cell lines from the CCLE (red) and 315 HGSOC tumor samples from TCGA (blue).

The 5000 most variable genes were used for unsupervised clustering of cell lines and tumors by mRNA expression data.

B. Principal component analysis of expression data from 47 ovarian cell lines from the CCLE (red) and 315 HGSOC tumor samples from TCGA (blue), using the 5000 most variable genes.

Figure S4

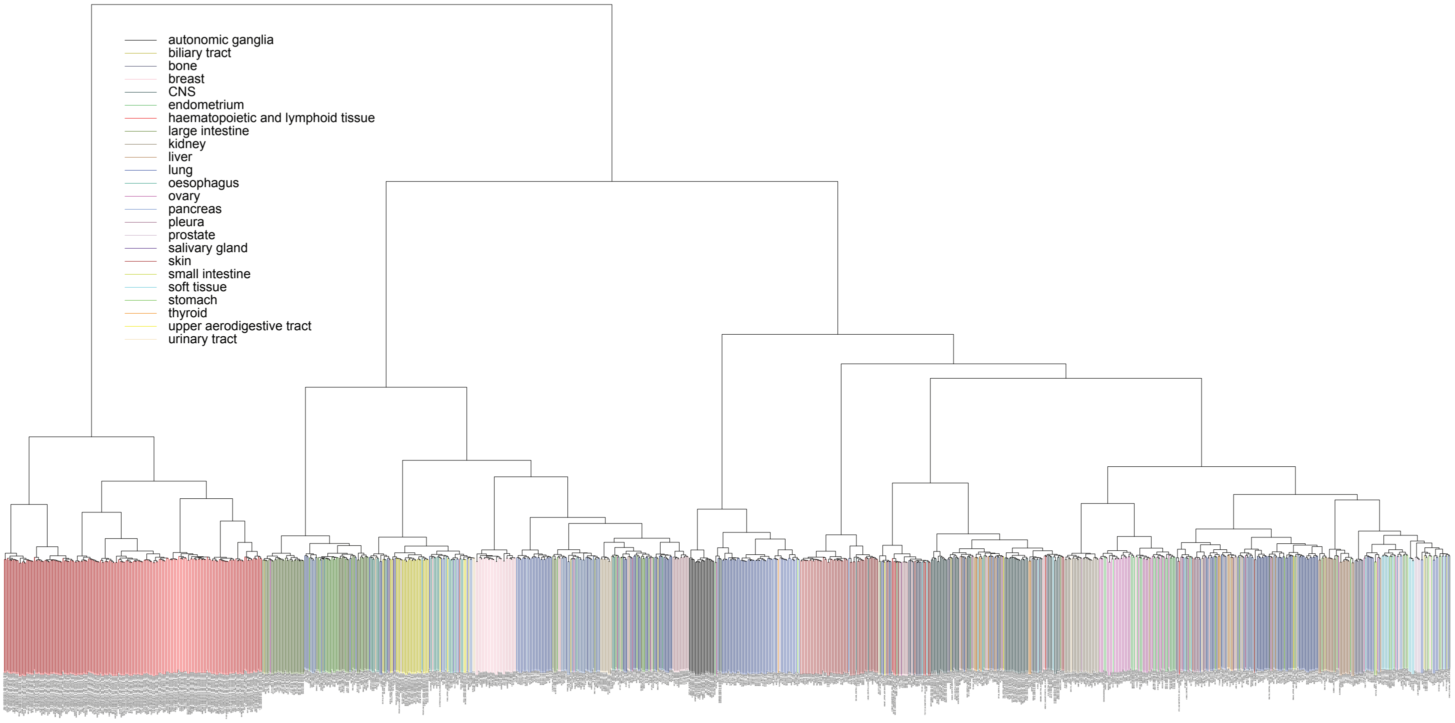

Figure S4. Expression-based clustering of all 963 CCLE cell lines across diverse tissue types - scalable graphic view.
The 5000 most variable genes were used for unsupervised clustering of cell lines by mRNA expression data. Cell lines are color-coded (vertical bars) according to the reported tissue of origin.
